# Changing ALK-TKI-Resistance Mechanisms in Rebiopsies of *ALK*-Rearranged NSCLC: *ALK*- and *BRAF*-Mutations Followed by Epithelial-Mesenchymal Transition

**DOI:** 10.3390/ijms21082847

**Published:** 2020-04-19

**Authors:** Edyta M. Urbanska, Jens B. Sørensen, Linea C. Melchior, Junia C. Costa, Eric Santoni-Rugiu

**Affiliations:** 1 Department of Oncology, Rigshospitalet, Copenhagen University Hospital, DK-2100 Copenhagen, Denmark; Jens.Benn.Soerensen@regionh.dk; 2 Department of Pathology, Rigshospitalet, Copenhagen University Hospital, DK-2100 Copenhagen, Denmark; Linea.Cecilie.Melchior@regionh.dk; 3 Department of Radiology, Rigshospitalet, Copenhagen University Hospital, DK-2100 Copenhagen, Denmark; Junia.Cardoso.Costa@regionh.dk; 4 Biotech Research & Innovation Centre (BRIC), University of Copenhagen, DK-2200 Copenhagen, Denmark

**Keywords:** ALK-rearranged NSCLC, crizotinib, ceritinib, alectinib, lorlatinib, ALK-TKI resistance, BRAF-mutation, EMT

## Abstract

*Anaplastic lymphoma-kinase (ALK)*-rearranged non-small cell lung cancer (NSCLC) is prone to developing heterogeneous, only partly known mechanisms of resistance to ALK-tyrosine-kinase-inhibitors (ALK-TKIs). We present a case of a 38-year old male, who never smoked with disseminated *ALK*-rearranged (*EML4* (20) – *ALK* (20) fusion variant 2) lung adenocarcinoma, who received four sequentially different ALK-TKIs and two lines of chemotherapy in-between. We observed significant clinical benefit by the first three ALK-TKIs (Crizotinib, Ceritinib, Alectinib) and chemotherapy with Pemetrexed, resulting in overall survival over 3 years. Longitudinal assessment of progressions by rebiopsies from hepatic metastases showed different mechanisms of resistance to each ALK-TKI, including secondary *ALK*-mutations and the downstream p.V600E *BRAF*-mutation that had not been linked to second-generation ALK-TKIs before. Ultimately, in connection with terminal rapid progression and resistance to Alectinib and Lorlatinib, we identified phenotypical epithelial-mesenchymal transition (EMT) of newly occurred metastatic cells, a phenomenon not previously related to these two ALK-TKIs. This resistance heterogeneity suggests a continuously changing disease state. Sequential use of different generation’s ALK-TKIs and combination therapies may yield prolonged responses with satisfactory quality of life in patients with advanced *ALK*-positive NSCLC. However, the development of EMT is a major hurdle and may explain rapid disease progression and lack of response to continued ALK-inhibition.

## 1. Introduction

Oncogenic fusion of the *anaplastic lymphoma-kinase* (*ALK*) gene is detectable in approximately 2%–7% of patients with non-small cell lung cancer (NSCLC), who are typically non-smokers and display adenocarcinoma histology. Most cases of NSCLC harboring *ALK*-rearrangements obtain clinical benefit from ALK-tyrosine-kinase-inhibitors (ALK-TKIs). However, despite long-lasting remissions with median overall survival (OS) of 81 months achieved by sequential ALK-TKIs, resistance inevitably appears in alternative dominating forms [1]. The baseline biology of *ALK*-positive NSCLC defined by the *echinoderm microtubule-associated protein-like 4 (EML4)-ALK* fusion variants, fusion partners (other than *EML4*), and other possible co-existing driver-mutations can affect the response to ALK-TKIs [1,2,3,4]. Conversely, resistance mechanisms may also vary and become more intricate when using second- or third-generation ALK-TKIs [2,5]. ALK-dependent resistance due to secondary mutations in the *ALK* TK-domain or gene copy number gain occurs more frequently with variant 3 of *EML4-ALK* rearrangement and upon treatment with next-generation ALK-TKIs [2,3,4,5]. There is also a correlation between multiple-line treatment with ALK-TKIs (≥ 2) and higher incidence of multiple mutations in the *ALK* gene as well as more frequent activation of bypass-signaling observed at rebiopsies [6]. Occasional cases of phenotypic transformation of *ALK*-positive NSCLC to neuroendocrine carcinoma after Lorlatinib or epithelial-mesenchymal transition (EMT) after treatment with Ceritinib have been reported [5,7]. Similarly, the EMT of *ALK*-positive NSCLC cell lines acquiring resistance to Crizotinib, Ceritinib or Brigatinib in vitro has been described [5,8,9,10]. These observations suggest that these phenotypic changes of tumor cells may represent additional mechanisms of resistance to ALK-TKIs. However, type and timing of resistance to ALK-TKIs cannot be foreseen in vivo and whether different mechanisms may subsequently/simultaneously occur during TKI-treatment in the same patient is still not clear.

## 2. Results

**Figure 1:**
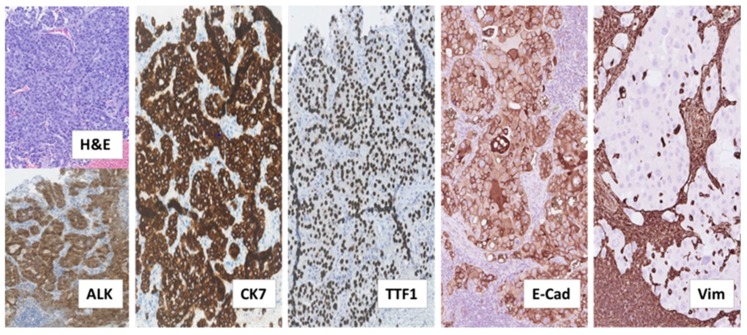
Histological examination of diagnostic biopsy from NSCLC metastasis to cervical lymph node. Tissue sections stained by hematoxylin and eosin (H&E) and the indicated immunostainings showed metastatic adenocarcinoma with tumor cells arranged in acinar, trabecular and solid structures. The tumor cells expressed the anaplastic lymphoma-kinase (ALK), consistent with *ALK*-rearrangement, as well as the adenocarcinoma marker Cytokeratin 7 (CK7) and the pulmonary marker Thyroid Transcription Factor 1 (TTF1). Additional immunostainings on deeper serial sections showed that the tumor cells expressed the epithelial marker, adhesion molecule E-Cadherin (E-Cad) and lacked expression of the mesenchymal marker Vimentin (Vim). In contrast, lymphocytes and histiocytes of the lymph node, including those infiltrating the tumor tissue did not express E-Cadherin and were positive for Vimentin. (All images: original magnification 100×).

The patient, a 38-year old, who never smoked, and was previously healthy, Caucasian male, was diagnosed with *ALK*-rearranged NSCLC in stage IV after examining a formalin-fixed paraffin-embedded (FFPE) biopsy taken from an enlarged cervical lymph node. This biopsy showed metastatic adenocarcinoma with acinar, trabecular and solid growth pattern that when assessed by immunohistochemistry (IHC) expressed Cytokeratin 7 (CK7), the thyroid/pulmonary biomarker Thyroid Transcription Factor 1 (TTF1), and ALK, consistent with rearrangement of the *ALK* gene (Figure 1).

Analysis of the tumor tissue by fluorescence in situ hybridization (FISH) displayed *EML4-ALK* rearrangement (70% of analyzed tumor cells), which was further confirmed by Archer^®^ anchored multiplex PCR (AMP™)/next-generation sequencing (NGS) assay performed on RNA isolated from the biopsy, demonstrating the fusion variant 2 (“long fusion”) between *EML4*-exon 20 and *ALK*-exon 20. Targeted NGS of the corresponding genomic DNA revealed no relevant single nucleotide variants (SNVs), indels or copy number variations (CNVs) across 52 genes analyzed by the utilized panel.

**Figure 2:**
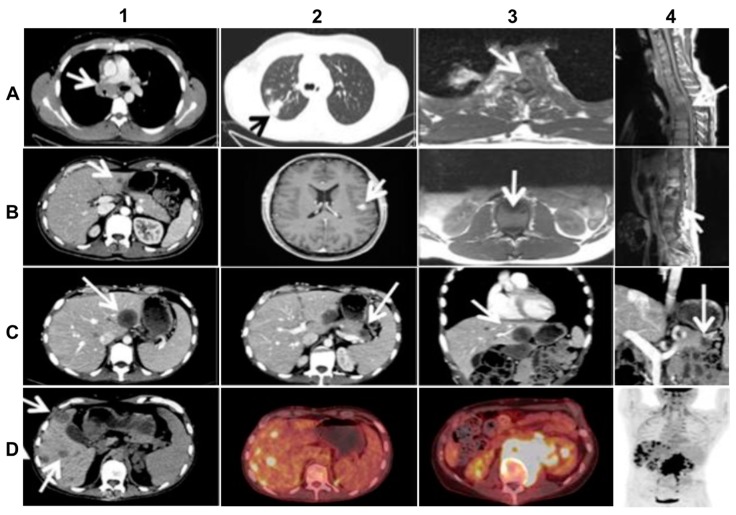
Imaging of the advanced (stage IV) *ALK*-rearranged NSCLC at diagnosis and at relapse after sequential treatment with Crizotinib, Alectinib, and re-challenge with Alectinib (*relevant changes indicated by arrows*). (**A**) top line from left to right: diagnostic CT (A1,A2) and MR (A3,A4) scans at baseline showing metastasized mediastinal lymph nodes compressing the vena cava superior (A1), the primary lung tumor in the right upper lobe (A2), transversal (A3) and sagittal (A4) images of vertebral metastases with spinal cord affection; (**B**) second line from left to right: CT (B1,B2) and MR (B3,B4) scans at progression after 10 months of Crizotinib treatment, showing liver metastasis (B1), the largest brain metastasis (B2), transversal (B3) and sagittal (B4) images of new vertebral metastases affecting the spinal cord; (**C**) third line from left to right: CT scans (C1–4) after 3 months of Alectinib treatment, exhibiting mixed response including new hepatic (C1,C3) and pancreatic (C2,C4) metastases on transversal and frontal planes; (**D**) last line from left to right: CT-(D1), PET/CT- (D2, D3), and upper part of total-body PET-scan (D4) at progression after re-challenge with Alectinib, showing multiple new hepatic metastases (D1,D2,D4) and a large retroperitoneal conglomerate of metastasized lymph nodes involving the left kidney (D3, D4).

The primary tumor was localized in the upper lobe of the right lung and was accompanied by multiple lymph nodal and vertebral metastases (Figure 2A). Because of a very poor clinical condition (PS 3) caused by transverse spinal cord syndrome and vena cava superior syndrome, the patient initially received salvage radiation against C7, Th3–Th5, Th12-L1 and the mediastinum. The patient improved quite rapidly after irradiation and initiated first-line treatment with Crizotinib 250 mg b.i.d. and Denosumab 120 mg s.c. every 4 weeks. After 10 months of good objective response to Crizotinib, which allowed the patient to partially resume work, the NSCLC progressed with new metastatic spinal cord compression and liver metastasis (Figure 2B). The first rebiopsy, taken from the new hepatic metastasis showed the occurrence of the p.C1156Y Crizotinib-resistant *ALK*-mutation (allele frequency (AF) = 15%). Because other ALK-TKIs were not available in Denmark at that time, the patient received second-line Cisplatin/Vinorelbine (Cis/Vin) chemotherapy and spinal cord irradiation against L3–S2. However, after two cycles of Cis/Vin chemotherapy, the cancer progressed further with multiple, very small, widespread metastases in the brain, the largest measuring one centimeter (Figure 2B), as well as a metastatic lesion in the right orbit causing abducens paresis and retinal detachment. After whole brain irradiation, the patient received third line systemic therapy with the next-generation ALK-TKI Ceritinib. After eight months with partial response and symptom-relief, the malignancy progressed again with new metastases to vertebrae, resulting in epidural protrusion, and to the liver and pancreas, while the patient’s general condition deteriorated to PS 3. As no additional irradiation could be given the patient started steroids, improving to PS 2. The second rebiopsy was taken from a new Ceritinib-resistant hepatic metastasis and displayed not only the persistence of the *EML4-ALK* fusion with p.C1156Y mutation in the ALK TK-domain (AF = 6%), but also the newly emerged p.D1203N *ALK*-mutation (AF = 9%) and p.V600E *BRAF*-mutation (AF = 12%). Each of the two *ALK*--mutations is supposed to be sensitive to the second-generation, highly CNS-penetrant ALK-TKI, Alectinib [2,3,5,11], while BRAF and MEK inhibitors were unavailable at our institution at the time. Therefore, given also the wide dissemination in the CNS, the therapy was changed to fourth line Alectinib, which again allowed the patient to clinically recover from PS 2 to PS 0 and return to work.

**Figure 3:**
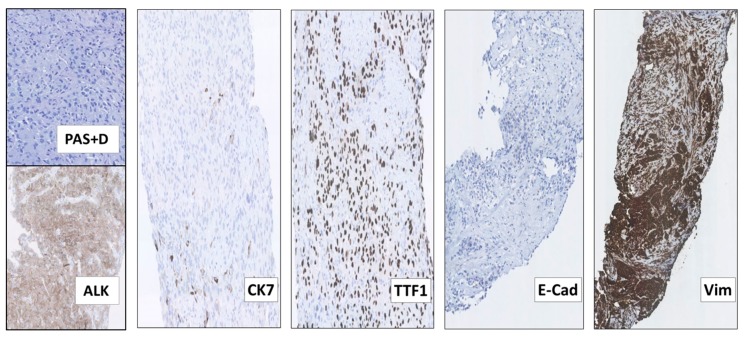
The 4th rebiopsy from the retroperitoneal NSCLC metastasis at progression after re-challenge with Alectinib, displaying features of epithelial-mesenchymal transition (EMT). Although many of the metastatic NSCLC cells had become more spindle-shaped and did not display any production of periodic acid–Schiff-positive diastase-resistant mucin (PAS+D), they still expressed the ALK fusion-protein (ALK). Most of the tumor cells had also lost the expression of the adenocarcinoma marker CK7, but maintained that of TTF1, consistent with their pulmonary origin. Moreover, the metastatic cells completely lacked the expression of the epithelial marker E-Cadherin (E-Cad) and had acquired that of the mesenchymal marker Vimentin (Vim), consistent with EMT. (All images: original magnification 100× except Vim 63×).

Following 3 months of Alectinib treatment a new CT scan revealed a mixed response with regression of two liver metastases and the appearance of other hepatic and pancreatic lesions (Figure 2C). Moreover, ophthalmological examination revealed a new choroidal metastasis in the right eye. The third rebiopsy taken from a progressing hepatic lesion 4 months after Alectinib was started, revealed retained *EML4-ALK* fusion without any *ALK*-mutations, but persistent p.V600E *BRAF*-mutation (AF = 7%). The *BRAF*-mutation, but no *ALK*-mutations, were also detected in the corresponding circulating-free DNA (cfDNA) from plasma (AF = 0,23%), further supporting a possible role in Alectinib-resistance. There were no reports of standard chemotherapy + ALK-TKI combination at that time, hence Alectinib was provisionally paused and the patient was started on fifth line Pemetrexed 500 mg/m^2^ IV every three weeks. Following six cycles of Pemetrexed with an initial partial response of some hepatic metastases, we observed stable disease in the bones and CNS, but progression of the primary tumor in the right lung. The latter was assumed to be caused by lack of ALK-inhibition. Thus, re-challenge with Alectinib was initiated together with Pemetrexed continuation. However, after 2 months of this combination treatment, creatinine and ALT/AST raised to grade 2–3 according to common terminology criteria for adverse events (CTCAE, v5.0), suggesting toxicity of the combined regime. Pemetrexed was stopped and the patient continued Alectinib only. After 3 months of re-challenge with Alectinib the PET/CT scan showed further occurrence of liver metastases and the emergence of a new 8.6 cm-large retroperitoneal lesion (Figure 2D). Other tumor components, including metastases in the CNS, were stable and no choroidal metastasis recurrence was detected. Alectinib was discontinued and a fourth tumor rebiopsy taken from the metastatic retroperitoneal conglomerate revealed still the persistence of *EML4-ALK* fusion, but no *ALK*-mutations and disappearance of the *BRAF*-mutation. A concomitant liquid biopsy did not show any DNA-mutations either. However, as shown in Figure 3, the retroperitoneal metastasis was poorly differentiated with solid arrangement of tumor cells not displaying any production of mucin, though still expressing the ALK fusion-protein. Indeed, we confirmed by NGS analysis of RNA that the retroperitoneal metastasis had retained the initial *EML4-ALK* fusion variant. Moreover, the metastatic cells had almost completely lost the expression of the adenocarcinoma-marker CK7 and despite maintaining the expression of the pulmonary marker TTF1, they tended to be spindle-shaped, lacked expression of the epithelial marker E-Cadherin and were strongly positive for the mesenchymal marker Vimentin (Figure 3).

These findings indicated that the retroperitoneal metastatic tissue (Figure 3), as compared to the adenocarcinoma tissue examined at baseline (Figure 1), had undergone phenotypical changes related to the EMT. This was further supported by supplementary IHC analysis revealing that the initial baseline metastasis in the cervical lymph nodes had preserved E-Cadherin expression and lacked Vimentin expression (Figure 1). Thus, features of EMT were not already present in the baseline tumor tissue. Similarly, we did not find phenotypic changes consistent with EMT in the first, second or third tumor rebiopsy.

Meanwhile, sixth line treatment with the third-generation ALK-TKI, Lorlatinib, was initiated, but the patient did not respond to the treatment and passed away 3 weeks later.

## 3. Discussion

**Figure 4:**
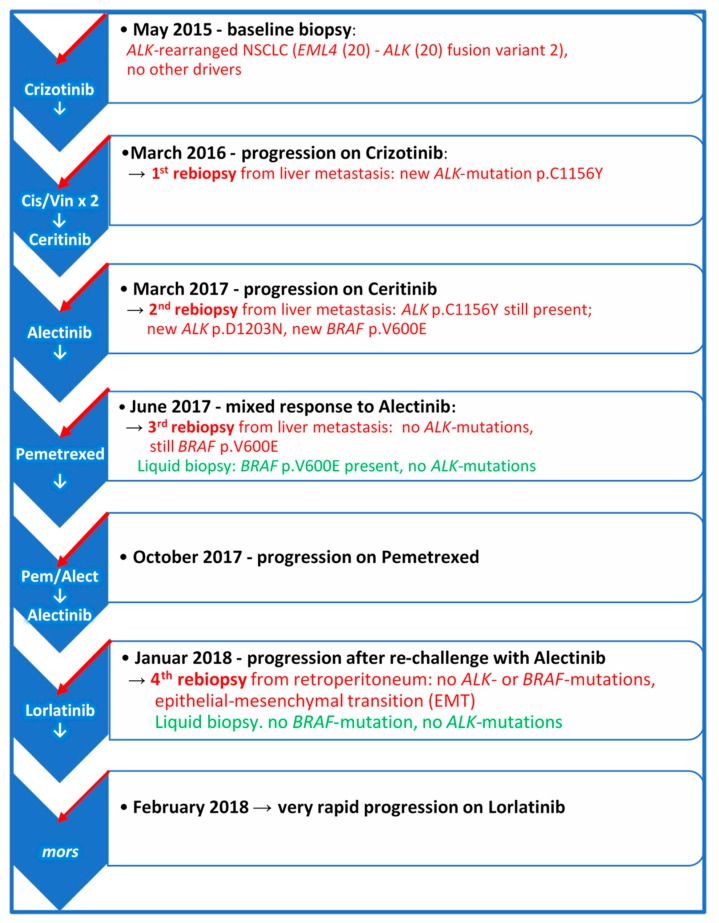
The course of the disease and systemic therapy from baseline at diagnosis to final progression. The scheme illustrates the molecular findings in the rebiopsies taken from new emerging metastatic sites and plasma at the time of progression on the indicated treatments. Cis/Vin × 2 = Two cycles of Cisplatin/Vinorelbine.

*ALK-*positive NSCLC is a very heterogenous disease which may progress through different molecular and phenotypic changes, so that despite frequent long-lasting objective responses to ALK-TKIs, resistance to these drugs inevitably occurs in different forms [1,2]. Progression patterns may be variable and may also depend on which generation of ALK-TKI has been used. Moreover, the biology of *ALK*-rearrangement plays an important role [1,2,5,6]. Despite developing more frequently TKI-resistant *ALK*-mutations, patients with *EML4-ALK* variant 3a/b may achieve longer PFS when treated with Lorlatinib as compared to patients carrying *EML4-ALK* variant 1 [4]. On the other hand, the incidence of *ALK* resistance mutations appears to increase with each successive generation of ALK-TKIs [11]. Figure 4 illustrates the longitudinal disease course and systemic treatment of the patient. In the first rebiopsy under progression on Crizotinib, we observed the emergence of p.C1156Y *ALK*-mutation, which alters the conformation of the ALK-binding pocket residues and results in a marked decrease in hydrogen bond interactions between Crizotinib and ALK fusion-protein. This mutation is supposed to be sensitive to Ceritinib. Nonetheless, the second rebiopsy, taken from new progressive liver metastasis after Ceritinib treatment, displayed the persistence of p.C1156Y and a new p.D1203N *ALK*-mutation, which is rare and had thus far been reported only in single patients relapsing on Ceritinib or Brigatinib, but not Alectinib [5,11]. However, p.D1203N is more common at relapse on Lorlatinib, almost always as compound mutation in combination with other *ALK*-mutants, such as p.I1171X, p.F1174X, p.L1196M, p.E1210K or p.G1269A, rather than on its own as a single mutation [2,5,11].

Importantly, we identified also a concomitant p.V600E *BRAF*-mutation at relapse on Ceritinib. *BRAF*-mutations have previously been detected in *ALK*-positive NSCLC patients after Crizotinib-treatment of [12], but to our knowledge this is the first report on the emergence of a *BRAF*-mutation during treatment with ALK-TKIs of second generation. Together, these observations indicate that *BRAF*-mutations such as p.V600E may represent a potential ALK-independent mechanism of resistance to ALK-TKIs, given the downstream placement of BRAF in the ALK-KRAS-MAPK signaling pathway. We could not exclude that what appeared to be the acquisition of mutated *BRAF* as a resistance mechanism in our patient, was a selection of a pre-existing p.V600E *BRAF*-clone by ALK-TKI treatment. Regardless of whether the *BRAF*-mutation represented a mechanism of acquired resistance or resulted from the selection of intrinsically ALK-TKI-tolerant cells [13,14], it is interesting that the third rebiopsy from a progressing hepatic metastasis taken 4 months after Alectinib-start retained the *EML4-ALK* fusion and the p.V600E *BRAF*-mutation, while p.C1156Y and p.D1203N *ALK*-mutations disappeared. The fact that p.V600E *BRAF* mutation was found in two subsequent tissue rebiopsies at progression on two different ALK-TKIs and in the following plasma cfDNA, supports its role in antagonizing ALK-inhibition. Unfortunately, neither the selective BRAF-inhibitor Dabrafenib, nor the MEK-inhibitor Trametinib were available at our institution at that time. Therefore, neither selective inhibition of the MAPK pathway with the Dabrafenib-Trametinib combination [15] nor combined anti-ALK/BRAF therapy could be attempted. In this regard, preclinical studies have provided a solid foundation for polytherapy with ALK-TKI combined with the MEK-TKI, Trametinib [16]. Preliminary results in the ongoing phase I/II study of Ceritinib + Trametinib (NCT03087448) so far are showing the feasibility of this combination with acceptable toxicity at reduced doses of both TKIs.

The last rebiopsy after progression on Pemetrexed and Alectinib re-challenge revealed the maintenance of the *EML4-ALK* fusion, although no *ALK-* or *BRAF*-mutations were detected in this tissue sample or in the concomitant plasma cfDNA. Considering that the *BRAF*-mutation was identified in the previous rebiopsies from hepatic biopsies, we cannot exclude that Pemetrexed caused or contributed to the disappearance of the *BRAF*-mutated tumor clone, as we did observe a partial response of some hepatic lesions to this drug. Alternatively, a form of synthetic lethal phenotype upon the arrest of Alectinib, leading to excessive MAPK signalling and toxic consequences in tumor cells co-expressing *ALK-*fusion and *BRAF*-mutation, may have occurred. Indeed, such an oncogene-induced toxic phenotype has been described in preclinical models of lung adenocarcinoma co-expressing mutations in *KRAS* and *EGFR* or *KRAS* and *BRAF* [17,18,19]. 

The mechanism of resistance to Alectinib at this time and the lack of response to the following attempted treatment with Lorlatinib may be associated with the observed phenotypical changes related to the EMT of the metastatic cells. Interestingly, EMT was previously described in a few patients at progression on Ceritinib [5] and in *ALK*-positive NSCLC cell lines becoming resistant to Crizotinib, Ceritinib or Brigatinib after prolonged exposure to these drugs [5,8,9,10], but to the best of our knowledge it has not been reported before in connection with Alectinib treatment of patients with *ALK*-positive NSCLC.

Secondary mutations in the *ALK* TK-domain that sterically impede the TKI-binding to the ALK fusion-protein are a common on-target mechanism of resistance to ALK-TKIs [1,2,5,6,11]. Each ALK-TKI appears associated with a distinct spectrum of ALK resistance mutations, though the solvent-front p.G1202R mutation that typically occurs after prolonged treatment with second-generation TKIs is resistant to all ALK-TKIs of first- and second-generation [2,5,6,11]. The current availability of several types of next-generation ALK-TKIs allows sequential treatment of patients with advanced *ALK*-positive NSCLC, choosing these drugs not only according to their potency and activity against the occurring secondary *ALK*-mutations, but also based on CNS-penetrance and possible side effects. Despite the clinical benefit from this approach, the selective pressure of sequential ALK-TKIs increases the likelihood of cancer cells developing new resistance mechanisms, such as different compound *ALK*- mutations or ALK-independent mechanisms, which almost always are refractory to currently available ALK TKIs [6,11]. The currently ongoing phase III clinical trial with Lorlatinib against advanced *ALK*-rearranged NSCLC will clarify the optimal sequential use of ALK-TKIs and whether it is possible to adopt Lorlatinib (hitherto, the ALK-TKI with the widest range of activity against single *ALK* resistance-mutations) as first line therapy, to avoid or reduce the occurrence of compound *ALK*-mutations. Alternatively, a fourth-generation ALK-TKI will be necessary to overcome the compound *ALK-*mutants [11].

Yet, a substantial number of NSCLC-patients receiving ALK-TKIs become resistant to these drugs through ALK-independent mechanisms [2,5,6,11]. The latter are only partially elucidated and particularly challenging, as they may inevitably cause refractoriness to further ALK inhibition, thereby requiring alternative forms of treatment to be counteracted. As in other reported cases of advanced *ALK*-positive NSCLC [5,6,11], we observed in the longitudinal re-biopsies taken at tumor progression during treatment the emergence of both ALK-dependent and ALK-independent mechanisms of ALK-TKI-resistance. Co-existence of the two types of resistance mechanisms can be detected in certain metastatic lesions, as indicated by our second re-biopsy at relapse on Ceritinib displaying secondary *ALK*-mutations and *BRAF*-mutation. This suggests the concomitant clonal evolution of different drivers of TKI-resistance within the same metastatic lesion. Importantly, the *BRAF* p.V600E mutation that for the first time we report emerging during patient treatment by second-generation ALK-TKIs, can potentially activate a bypass signaling downstream ALK that causes refractoriness to ALK-TKIs. Thus, the occurrence of this *BRAF*-mutation at progression on ALK-TKIs may represent an indication for combined targeted therapy with currently available ALK- and BRAF/MEK-inhibitors.

Moreover, we showed for the first time the occurrence of EMT in advanced *ALK*-positive NSCLC upon progression on Alectinib. The EMT was not only associated with refractoriness to Alectinib, but also with total lack of response to the following therapeutic attempt with Lorlatinib, indicating that potent ALK inhibition was no longer effective in this situation. As described in a few cases of Ceritinib-resistant *ALK*-positive NSCLC [5], the EMT is characterized by loss of epithelial markers, particularly of the cell-adhesion protein E-Cadherin, by the NSCLC cells, which acquire mesenchymal phenotypical features and become spindle-shaped, overexpress Vimentin, increase their motility and invasiveness, and ultimately turn into TKI-resistant cells. Several transcription factors and related genes have been implicated in the induction of EMT by TKI-treatment, however it is still poorly understood how EMT causes TKI-resistance [13,14]. Indeed, a key event in the induction of TKI-resistance in the NSCLC cells appears to be the downregulation of E-Cadherin itself [13,14], as also suggested by recently reported mutations of genes regulating EMT and E-Cadherin expression in Crizotinib-resistant NSCLC [20]. Furthermore, EMT may cause TKI-resistance at least in part by transcriptional downregulation of BIM, a pro-apoptotic Bcl-2 family member required for TKI-induced apoptosis of NSCLC cells [21]. Preclinical studies in NSCLC cell lines have also shown that TKI-treatment can induce overexpression of the receptor-TK AXL, which contributes to the EMT of these cells allowing them to survive the treatment and proliferate [9]. Finally, an intricate interplay between cancer hypoxia and EMT-induction resulting in the activation of insulin-like growth factor 1 receptor (IGF1R) and TKI-resistance has been postulated in NSCLC [14,22]. Notably, EMT was recently observed in two patient-derived *ALK*-positive NSCLC cell lines becoming resistant to Lorlatinib [23]. Moreover, concomitance of cells with *ALK*-mutation L1196M and cells with EMT as independent mechanisms of Crizotinib-resistance have been described in a patient with *ALK*-rearranged NSCLC [24]. Additionally, in preclinical experiments with *ALK*-positive NSCLC cells becoming refractory to Crizotinib, the occurrence of EMT caused cross-resistance to the ALK-TKIs of new-generation Ceritinib, Alectinib, and Lorlatinib [24]. Together with our case, these observations support the notion that the EMT may counteract the effects of all the ALK-TKIs currently used in the clinics and further explain the lack of response of our patient to Lorlatinib. 

Further characterization of *ALK*-rearranged NSCLC patients participating in clinical trials or from real-life cohorts will likely provide additional information on the potential ALK-dependent/-independent mechanisms of resistance to different ALK-TKIs. As suggested by this and previous reports [2,5,6,11], comprehensive longitudinal monitoring of these patients through sequential tissue and liquid re-biopsies taken at relapses during treatment is necessary for this purpose. This general approach will also help decipher the frequency and impact of *BRAF*-mutations as critical bypass signaling potentially involved in ALK-TKI resistance. Moreover, it will elucidate the mechanisms by which EMT causes TKI-resistance.

## 4. Materials and Methods 

### 4.1. Immunohistochemistry (IHC) and Histochemical Stain

IHC was performed on FFPE 2.5-μm-thick tissue sections using a BenchMark ULTRA automated slide immunostainer (Ventana Medical Systems, Inc., Roche Diagnostics; Hvidovre, Denmark). The pre-diluted Ventana’s rabbit mAbs D5F3 against ALK, SP52 against CK7, SP141 against TTF1, and EP700Y against E-Cadherin as well as the mouse mAb 3B4 against Vimentin (Ventana, Roche Diagnostics; Hvidovre, Denmark), were employed according to the manufacturer’s instructions and staining conditions, including the usage of the corresponding negative control (sections stained with an unrelated matched rabbit IgG mAb) and the positive control (samples with *ALK*-rearrangement or known expression of the specified antigens), as previously described [25,26]. The histochemical stain for mucin was performed by standard periodic acid–Schiff + diastase digestion (PAS+D) method.

### 4.2. Fluorescence in situ Hybridization (FISH)

FISH analysis of *ALK*-rearrangement in tumor cells was performed utilizing the triple color ZytoLight^®^ SPEC ALK/EML4 TriCheckTM probe (Zytovision GmbH, AH diagnostics A/S, Tilst, Denmark), according to principles described before [25], with minor modifications. Briefly, 1.5-μm-thick sections were scanned using a X63 objective and appropriate filter sets in an automated Leica DM5500 B fluorescent microscope (Leica MICROSYSTEMS A/S; Copenhagen, Denmark), using fibroblasts, leukocytes, and endothelial cells as internal controls and individually assessing 100 tumor cell nuclei (20 neighboring tumor cell nuclei from five random areas of homogenous distribution of *ALK* signals) for *ALK* (green and orange) and *EML4* (blue) signals with the X100 objective. Cut-off for positive rearrangement was 15%.

### 4.3. Multiplex PCR/NGS Assay for Gene Fusions

To confirm the *EML4-ALK* fusion and specify the fusion variant, Archer^®^ anchored multiplex PCR (AMP™)/NGS assay (FusionPlex^®^ Solid Tumor Kit) was performed on RNA isolated from tissue biopsies, according to the manufacturer’s instructions (ArcherDX, Inc., Boulder, CO, USA).

### 4.4. Analysis of Mutations in Tumor-Rebiopsies and Liquid Biopsies 

To identify TKI-resistance mechanisms during treatment, the baseline biopsy and four longitudinal re-biopsies from new consecutive metastatic lesions emerging during the disease evolution were analyzed histologically and by IHC for ALK-protein expression, FISH and NGS for *ALK*-rearrangement and other gene fusions. Moreover, targeted NGS of DNA was performed for hot-spot mutations such as relevant SNVs, indels and CNVs across 52 genes in the panel, according to the assay’s instructions (Oncomine Focus Assay; ThermoFisher Scientific, Roskilde, Denmark). For each FFPE biopsy, 10 ng of genomic DNA, purified by the QIAamp DNA Minikit (QiagenAB, Vedbæk, Denmark) and quantified by the Qubit^®^ dsDNA HS assay on a Qubit^®^ 2.0 Fluorometer (ThermoFisher Scientific, Roskilde, Denmark), were used. After preparation of amplicon-based libraries, the DNA was sequenced on the Ion Torrent™ GeneStudio™ S5 Plus System (ThermoFisher Scientific, Roskilde, Denmark) according to the manufacturer’s instructions. Additionally, liquid biopsies (cfDNA from plasma) were analyzed for relevant DNA-mutations by the Oncomine Lung cfDNA NGS-assay according to the manufacturer’s instructions (ThermoFisher Scientific, Roskilde, Denmark).

### 4.5. Ethical Aspects

The patient’s family gave informed written consent for the publication of this paper, including clinical data and images of the deceased patient. The study was conducted in accordance with the Declaration of Helsinki and the ethical guidelines of the Capital Region of Denmark. The case was part of a clinical study on *ALK*-positive NSCLC patients at our institution that was approved by the local ethical committee of Rigshospitalet, Copenhagen University Hospital and by the Danish Capital Region’s Committee for Health Research Ethics (project identification code and approval number: H-15008619; approval date: 23 March 2017).

## 5. Conclusions

Following the treatment course of this disseminated *ALK*-rearranged NSCLC by longitudinal rebiopsy-based assessment, we observed heterogeneous and transitional mechanisms of resistance to different ALK-TKIs. These included the emergence of secondary *ALK*-mutations and the p.V600E *BRAF*-mutation, which had not previously been associated with resistance to Ceritinib and Alectinib. In addition, we detected phenotypical changes consistent with EMT following treatment with Alectinib. EMT of *ALK*-positive NSCLC related to ALK-TKI treatment remains poorly explored and to our knowledge it has not been reported before in patients receiving Alectinib, but only in a few patients progressing on Crizotinib or Ceritinib [5,24]. This resistance heterogeneity suggests a continuously evolving state of the disease. Sequential use of different generation’s ALK-TKIs and/or combination therapies may allow prolonged responses (over 3 years in this case) with a satisfactory quality of life. However, phenotypical EMT-related changes represent a major hurdle for TKI-based therapy and, as in our case, may explain rapid disease progression and a lack of response to second- and third-generation ALK-TKIs, despite the preserved *ALK*-positive status of the tumor. Thus, this case illustrates that *ALK*-positive NSCLC is biologically a very unstable disease that changes its genetic makeup over time, while developing different forms of resistance.
